# Respiratory Diseases in University Students Associated with Exposure to Residential Dampness or Mold

**DOI:** 10.3390/ijerph13111154

**Published:** 2016-11-18

**Authors:** Mathieu Lanthier-Veilleux, Geneviève Baron, Mélissa Généreux

**Affiliations:** 1 Department of Community Health Sciences, Faculty of Medicine and Health Sciences, Université de Sherbrooke, Sherbrooke, QC J1H 5N4, Canada; Genevieve.baron@usherbrooke.ca (G.B.); melissa.genereux@usherbrooke.ca (M.G.); 2 Public Health Department of Eastern Townships, 300 King East, Sherbrooke, QC J1G 1B1, Canada

**Keywords:** dampness, mold, housing, university students, asthma, allergic rhinitis, respiratory infections

## Abstract

University students are frequently exposed to residential dampness or mold (i.e., visible mold, mold odor, dampness, or water leaks), a well-known contributor to asthma, allergic rhinitis, and respiratory infections. This study aims to: (a) describe the prevalence of these respiratory diseases among university students; and (b) examine the independent contribution of residential dampness or mold to these diseases. An online survey was conducted in March 2014 among the 26,676 students registered at the Université de Sherbrooke (Quebec, Canada). Validated questions and scores were used to assess self-reported respiratory diseases (i.e., asthma-like symptoms, allergic rhinitis, and respiratory infections), residential dampness or mold, and covariates (e.g., student characteristics). Using logistic regressions, the crude and adjusted odd ratios between residential dampness or mold and self-reported respiratory diseases were examined. Results from the participating students (*n* = 2097; response rate: 8.1%) showed high prevalence of allergic rhinitis (32.6%; 95% CI: 30.6–34.7), asthma-like symptoms (24.0%; 95% CI: 22.1–25.8) and respiratory infections (19.4%; 95% CI: 17.7–21.2). After adjustment, exposure to residential dampness or mold was associated with allergic rhinitis (OR: 1.25; 95% CI: 1.01–1.55) and asthma-like symptoms (OR: 1.70; 95% CI: 1.37–2.11), but not with respiratory infections (OR: 1.07; 95% CI: 0.85–1.36). Among symptomatic students, this exposure was also associated with uncontrolled and burdensome respiratory symptoms (*p* < 0.01). University students report a high prevalence of allergic rhinitis, asthma-like symptoms and respiratory infections. A common indoor hazard, residential dampness or mold, may play a role in increasing atopic respiratory diseases and their suboptimal control in young adults. These results emphasize the importance for public health organizations to tackle poor housing conditions, especially amongst university students who should be considered “at-risk”.

## 1. Introduction

Respiratory diseases can result in restricted activity, sick leave, and hospitalization [1,2,3], and cost more than eight billion CAD in direct and indirect costs annually in Canada alone [3]. While children and the elderly are widely recognized as vulnerable populations to respiratory diseases, many adults also suffer from allergic rhinitis, asthma [4], and respiratory infections [5]. In fact, young adults are more frequently affected by some respiratory diseases than children and the elderly, with symptoms of asthma and allergic rhinitis reaching their highest prevalence (6% and 22%) at 15–19 and 25–44 years of age, respectively [1,6]. Bacterial respiratory infections and recurrent viral respiratory infections are also very frequent in young adults [7,8].

Indoor air environment exposure is now recognized as an important respiratory health determinant worldwide and could be responsible for up to one-third of the burden associated with respiratory diseases [3,9,10,11]. Various mold species are well-established and prominent indoor air hazards which can contribute to both atopic (mainly allergic rhinitis and asthma) and infectious respiratory symptoms [9,12]. More specifically, exposure to dampness or mold has been associated with the development or exacerbation of common respiratory diseases such as asthma, allergic rhinitis, and respiratory infections, with odd ratios (OR) reaching 1.50 (95% confidence interval (CI): 1.25–1.80), 1.83 (95% CI: 1.75–1.91), and 1.49 (95% CI: 1.14–1.95), respectively [9,13,14]. It is estimated that roughly half of 20–34 years old asthmatic patients have symptoms that are triggered by exposure to dampness or mold [1].

Up to one-third of Canadian buildings present signs of dampness or mold [15,16]. Populations exposed to dampness or mold are characterized by low income, tenancy, and residential mobility, three factors that are common among university students [7,17]. Two recent North American studies conducted in Quebec (Canada) and in Utah (USA) revealed that more than one in three university students are exposed to dampness or mold (36% and 39%, respectively) [8,17].

Despite the high likelihood of exposure to dampness or mold among university students, and despite significant associations with respiratory health, very few studies have evaluated the contribution of indoor environmental factors such as dampness or mold to university students’ respiratory health [7,8]. Moreover, considering that many environmental (e.g., building characteristics, climate, air pollution) and individual (e.g., genetic profile, behavior) risk factors for respiratory diseases [18] are subject to important geographic variations, the evaluation of this exposure’s impact on respiratory health needs to be replicated in a North American context.

This study thus aims to: (a) describe the prevalence of asthma, allergic rhinitis, and respiratory infections among university students; and (b) examine the independent contribution of residential exposure to dampness or mold to these health issues and explore its impact on quality of life.

## 2. Materials and Methods

Materials and methods have been described in detail in a previously published article [17]. This section will summarize key elements of the methods, including questionnaire development, variables and statistical analyses that are specific to this paper.

### 2.1. Studied Population and Response Rate

This method has been described in detail elsewhere [17]. The study population consists of students registered at the Université de Sherbrooke during the 2014 winter trimester. Ethical and organizational approvals were obtained from the Centre de Recherche du CHUS research ethic board, from the Université de Sherbrooke and from student federations (project identification code for CHUS ethic board approval is 2014-752, 14-022). Students were contacted via their university email in early March 2014. To maximize response rate, many strategies proposed by Dillman [19,20] were applied as previously described [17]. When considering the target population (*n* = 26,676) and after correction for ineligibility among respondents (2.5%), the final participation rate was 8.1% (2097/26,009). Consequences of this low participation rate are discussed in the Limitations section of the Discussion.

### 2.2. Questionnaire Development

The survey questionnaire was developed using questions selected from validated questionnaires. The Grassi score [21], from the European Community Respiratory Health Survey (ECRHS), was used to evaluate asthma-like symptoms (sensitivity and specificity of 75% and 80%, respectively, when compared to a clinical diagnosis). The ECRHS includes questions about wheezing, shortness of breath, asthma crisis, and use of asthma drugs. The Score For Allergic Rhinitis (SFAR) score [22] was used to assess allergic rhinitis (sensitivity and specificity of 74% and 83%, respectively, when compared to a medical evaluation). It includes questions about ocular and nasal symptoms in the absence of respiratory infection, medical and laboratory diagnoses for allergic rhinitis, atopic triggers and parental atopy. We used criteria based on Kilpelainen et al. (described in the Variables section) to define significant respiratory infections [7]. While the validity of these criteria has not yet been evaluated, they allow for a better comparison of our results with Kilpelainen’s study. Atopy control was evaluated via two visual analog scales (VAS), which show good correlations with validated questionnaires (*p* = −0.70 for the Asthma Control Test questionnaire and *p* = 0.46 for the Rhinoconjonctivitis Quality of Life Questionnaire) [23,24], and via criteria based on the widely-recognized Global Initiative for Asthma (GINA) questionnaire [25,26]. The selected criteria (described in the Variables section) for residential exposure to dampness or mold have a sensitivity and specificity of 75% and 71%, respectively, when compared to a hygienist’s evaluation [27] and are derived from the ECRHS II questionnaire [28]. The questionnaire also included questions pertaining to socio-demographic factors or other covariates (intrinsic individual characteristics, student characteristics, socioeconomic factors, and other environmental exposures) and has been validated for content validity as well as pilot tested before its use.

### 2.3. Variables

As shown in our previously published conceptual model [17], three dependent variables were studied: asthma-like symptoms, allergic rhinitis, and respiratory infections. As atopy could be widely underdiagnosed [16,29], self-declared asthma-like symptoms, rather than physician-diagnosed asthma, were considered for the main analyses. Students were considered as having an atopic respiratory disease if they achieved a score ≥1 on the Grassi scale for asthma-like symptoms (i.e., affirmative answer to any significant questions) [21], or scored ≥7/16 on the SFAR score for allergic rhinitis. Atopy prevalence was also evaluated using other self-reported criteria: respondent’s perception of being allergic, clinical diagnosis of atopy by a physician, and positive skin test for specific environmental markers (house dust, house dust mites, pollen, animals, or mold). Physician-diagnosed asthma reported by respondents was used to estimate lifetime and active diagnosed asthma. As for respiratory infections, they were considered significant when respondents reported having had four or more viral infections (flus or colds), or at least one bacterial respiratory infection (defined as pneumonia, bronchitis, sinusitis, otitis media, or tonsillitis) that required antibiotic treatment, in the past year [7]. Most questions about symptom activity covered this time interval (i.e., the previous 12 months).

In the presence of active asthma-like symptoms or allergic rhinitis, questions about symptom management and impact on quality of life were asked as well as whether or not symptoms were interfering with academic results. Impact of symptoms on quality of life in the last three months was assessed using two VAS of 10 cm each, ranging from “not at all” (0) to “unbearable” (10). Active asthma-like symptoms or allergic rhinitis were considered as burdensome at VAS scores of ≥3. As for the level of control in symptom management, diagnosed active asthma was considered uncontrolled when the GINA criteria matched either the “uncontrolled” or “partially controlled” categories [26].

The independent variable, residential exposure to dampness or mold, was defined as at least one of the following four signs at the respondent’s term-time address in the past 12 months: (a) visible mold; (b) mold odor; (c) dampness such as wet or damp spots on surfaces; and (d) present or past water leaks not cleaned within 48 h (as per U.S. Environmental Protection Agency recommendations) [15,27]. Symptomatic students were asked to qualify their perception of a spatiotemporal link between their symptoms and their housing. Past exposure to insalubrious housing requiring a change of residence was also evaluated.

The following self-reported individual and environmental characteristics were examined as covariates in the study: (1) intrinsic individual characteristics such as sex, age, and parental atopy; (2) student characteristics such as campus location (used as a proxy for residential location, as the Université de Sherbrooke has campuses in various locations across the province of Quebec); (3) socioeconomic factors such as homeownership and family annual income, which included the student’s and, when relevant, the spouse’s income, as well as the income from any other source (e.g., family allowance); and, (4) other environmental exposures such as smoking status, passive smoking, pets, and carpets at home, as well as residential exposure to traffic-related air pollution assessed by using road density at the community level based on the respondent’s residential postal code.

### 2.4. Statistical Analysis

Frequency distributions and crosstables were used to describe the study sample and Chi-square (χ^2^) tests and univariate logistic regressions to measure crude associations. The difference in mean road density between healthy and symptomatic students was examined using student’s *t*-test. An alpha value of 0.05 was used for statistical significance. Only respondents with available weighting variables (sex, age, and campus affiliation) were included for bivariate analyses (*n* = 1971).

Multivariate logistic regressions were conducted using a step-by-step method with plausible confounders. In addition to age and sex, explanatory variables that were significantly associated with any of the three main respiratory diseases at an alpha value of 0.10 were included in the three models. In accordance with our conceptual model, parental atopy and smoking status were tested as potential moderators [7,30]. Variables considered as potential mediators were excluded since the aim of the analysis was to assess global (direct or indirect) associations between exposure and each respiratory disease individually (i.e., allergic rhinitis was excluded in the asthma-like symptoms model [31]).

Further exploratory analyses were undertaken among symptomatic students. Through multivariate modelling, associations between exposure to dampness or mold and exploratory dependent variables (burdensome symptoms, uncontrolled asthma, decrease in academic performance, and perceived spatiotemporal link with housing) were investigated. These models used fewer covariates because of decreased statistical power.

Weighting was not used in the multivariate analyses because it can mask possible interaction effects [32]. All analyses were performed using SPSS version 21 (IBM Corp., Armonk, NY, USA).

## 3. Results

### 3.1. Descriptive Statistics

A complete description of the socio-demographic and environmental characteristics of respondents has been previously published [17]. Comparison of the final sample (*n* = 2097) with the study population (*n* = 26,676) revealed that women (70.3% vs. 56.3% in the sample and in the study population, respectively), students from the main campus (65.5% vs. 54.1%), and younger students (55.3% vs. 40.1%) were significantly overrepresented in the final sample. Most respondents were born in Canada, spoke French at home and studied full time. Over half of participants were young students (18 to 23 years old), baccalaureates, and studied at the main campus. More than 80% were tenants and more than half (59%) declared a family annual income below CAD $25,000. Finally, residential exposure to dampness or mold was frequent (36.0%, 95% CI: 33.9–38.1).

### 3.2. Prevalence of Respiratory Diseases

Table 1 presents the prevalence of self-reported respiratory diseases (allergic rhinitis, asthma-like symptoms, and significant respiratory infections) and associated conditions such as atopy. The prevalence of atopy was greatly affected by the definition chosen. One out of three students considered themselves allergic (33.7%). However, when adding more objective criteria (i.e., physician diagnosis, positive skin test for classic environmental triggers and, specifically, for mold), this proportion decreased to 29.0%, 19.6%, and 5.6%, respectively. One-quarter of students reported active asthma-like symptoms in the last 12 months. Other respiratory diseases were also frequent, with one-third of students declaring having allergic rhinitis and one-fifth reporting significant respiratory infections. Overall, almost half (49.0%) of students had at least one of these three respiratory diseases, 16.6% had at least two and 2.5% reported all three diseases.

### 3.3. Bivariate Analyses by Disease

Table 2 characterizes the prevalence of self-reported respiratory diseases according to several factors. All three diseases were associated with sex, women constantly presenting a higher prevalence of disease than men (all *p* < 0.01). Asthma-like symptoms seemed to decrease with age, the OR between the youngest and oldest age groups being of 0.72 (95% CI: 0.56–0.92) while allergic rhinitis appeared to increase with age, with an OR between the same age groups of 1.28 (95% CI: 1.03–1.61). As expected, associations between parental atopy and both allergic rhinitis and asthma-like symptoms were observed, with OR of respectively 4.76 (95% CI: 3.83–5.91) and 1.87 (95% CI: 1.49–2.35). Parental atopy was also associated with respiratory infections, but somewhat less strongly, with an OR of 1.32 (95% CI: 1.03–1.70). No clear trend could be observed between annual family income and respiratory diseases, except for asthma. Indeed, students with higher income (over CAD $55,000) reported less asthma-like symptoms compared to the lowest income group (less than CAD $15,000), with an OR of 0.63 (95% CI: 0.48–0.84). Both former and current smokers had a higher prevalence of asthma-like symptoms, with OR of 1.46 (95% CI: 1.04–2.06) and 1.46 (95% CI: 1.08–1.99) respectively, while only former smokers had a higher prevalence of allergic rhinitis in comparison to non-smokers, with an OR of 1.44 (95% CI: 1.08–1.91). Interestingly, past experience of insalubrious housing was significantly associated with all three diseases, particularly for allergic rhinitis and asthma-like symptoms, with respective ORs of 2.97 (95% CI: 2.16–4.08) and 2.95 (95% CI: 2.14–4.07).

### 3.4. Multivariate and Exploratory Analyses

Table 3 presents bivariate and multivariate models estimating associations between residential exposure to dampness or mold and each of the three respiratory diseases. In bivariate analyses, allergic rhinitis and asthma-like symptoms were both significantly associated with exposure to dampness or mold (*p* < 0.01), but respiratory infections were not. After adjustment for covariates, allergic rhinitis and asthma-like symptoms were still significantly associated with this exposure with ORs of 1.25 (95% CI: 1.01–1.55) and 1.70 (95% CI: 1.37–2.11), respectively.

Exploratory analyses (Table 3) revealed that among students with allergic rhinitis, residential exposure to dampness or mold was strongly associated with burdensome symptoms (OR: 1.75; 95% CI: 1.22–2.51), as exposed students were almost twice as likely to report burdensome symptoms than unexposed students. Similarly, among students with asthma-like symptoms, those exposed were more likely than those unexposed to report burdensome and uncontrolled asthma (OR: 2.34; 95% CI: 1.31–4.16 and OR: 2.17; 95% CI: 1.21–3.88, respectively). Moreover, regardless of disease, exposed symptomatic students were more susceptible to attribute their symptoms to time spent at home (OR: 2.80; 95% CI: 1.93–4.06). Finally, among symptomatic students, residential exposure to dampness or mold was associated with a decrease in academic performance (6.5% vs. 10.6% among unexposed vs. exposed symptomatic students), even after controlling for covariates (OR: 1.79; 95% CI: 1.12–2.84).

No interaction was observed with smoking status or parental atopy. Exposure to pets and carpets was not associated with asthma or allergic rhinitis. Road density was not included in the multivariate logistic regression models as it was not associated with any respiratory disease, or their symptom management, in bivariate analyses.

## 4. Discussion

Our findings reveal a high prevalence of self-reported respiratory diseases in this population of North American university students as well as significant associations with residential exposure to mold or dampness.

The prevalence of respiratory diseases observed in the present study is coherent with previously reported data. Observed asthma-like symptom prevalence (24%) falls in the interval of wheezing prevalence among young Canadian adults 20 to 44 years of age (22% to 30%) [33]. Allergic rhinitis prevalence (33%) is similar to the prevalence reported by the French national survey INSTANT (31%) conducted on a population ranging from 20 to 44 years old using the same SFAR score as the present study [34]. On the other hand, the observed prevalence of significant viral and bacterial respiratory infections (12% and 10%, respectively) were much lower than those reported by Finnish university students (16% and 34% respectively) or by Minnesota college students (86% for at least one cold, 37% for at least one influenza-like illness, and 16% for antibiotics use) [7,35]. These major differences could be in part explained by how study outcomes were defined. For example, the Minnesota study used more sensitive definitions compared to both our study and the Finnish study (e.g., at least one viral infection vs. at least four episodes) [7,35]. Seasonal influenza activity could be another factor, since winter 2014 was a moderate season for influenza in Canada [36].

As expected, students with a family history of atopy presented a higher prevalence of atopic respiratory diseases, but not of respiratory infections. On the other hand, we could not replicate a previously-reported association between housing-related health issues and socioeconomic status [37]. The absence of a clear trend between respiratory symptoms and family income suggests the lack of a socioeconomic gradient in our young university student population, as was the case in a recent respiratory health study among children based in Montreal, Quebec [15]. That study found no difference in disease prevalence according to socioeconomic status when other variables were taken into account. While university students could be better equipped than other populations of low socioeconomic status at finding proper housing, further research could help better understand these counterintuitive results. Results showing a strong association between respiratory disease and past exposure to insalubrious housing may be explained by previously developed mold sensitivity or by some students’ difficulty to afford salubrious housing. Despite the absence of a socioeconomic gradient, our findings suggest that some symptomatic students may have challenges in moving away from their insalubrious environment [38].

Observed associations between residential exposure to dampness or mold and respiratory diseases are similar to those reported in two Finnish studies, including one conducted among university students [7,39]. However, the association previously found between this exposure and respiratory infections was not replicated in the current study despite sufficient statistical power. Nevertheless, our results went further than previous studies by suggesting that exposure to dampness or mold increases the students’ likelihood of suffering from burdensome and/or uncontrolled symptoms. These findings reinforce the importance, for health professionals, of asking their patients with suboptimal symptom control about their environmental exposures. Of concern, we demonstrated that residential exposure to dampness or mold was also associated with a perceived lower academic performance amongst symptomatic students. As with the previously-reported association between influenza-like illness and academic performance [35], these results support the hypothesis that frequent or severe respiratory symptoms could have a negative influence on academic results, but more research should be done to confirm this association.

Moreover, no interaction was established between exposure to residential mold or dampness and parental atopy. This is in contradiction with Kilpelainen et al. who reported that exposure to dampness or mold was associated with asthma and allergic rhinitis only among people with parental asthma or atopic disease [7]. Our findings rather reinforce the hypothesis that dampness in buildings affects both atopic and non-atopic populations as described in a previous multidisciplinary review [40]. Finally, little change in the magnitude of association was observed after adjustment for covariates, which supports an independent relation between exposure to dampness or mold and self-reported respiratory diseases.

### Limitations

As it is detailed in a previous article [17], this cross-sectional study has some limitations. An important one is the low response rate (8%). Web surveys tend to have much lower response rates than mail surveys [41]. For instance, a recent Quebec web survey among college students (18 to 24 years of age) had a 10% response rate [42] and another among Université de Sherbrooke students only reached 7% [43]. Nevertheless, university students are more easily reached this way, as all students have to regularly use their Université de Sherbrooke email account.

Non-response bias in this study may have led symptomatic students to self-select in greater proportion than healthy ones, leading to an over-estimation of the prevalence of respiratory disease. Results were weighted for key socio-demographic characteristics to correct for different sociodemographic groups’ participation rate. Moreover, it should be noted that comparable results regarding respiratory disease prevalence were reported in previous studies based on populations of the same age group and with higher response rates [7,33,34]. Despite these corrections and comparisons, the study’s prevalence estimates should nevertheless be interpreted with caution given the low participation rate. However, associations between dampness or mold and respiratory diseases should not be affected by this low response rate, assuming that the decision to participate in the study was independent of the associations being investigated.

To limit classification bias associated with self-reported data, we used, whenever possible, the best available and validated measures to assess our main dependent and independent variables. As presented in Table 1, our findings highlight the importance of variable definition, as self-declared symptoms or diseases were often not diagnosed by a medical doctor [16,29]. Asthma and allergic rhinitis may be underdiagnosed, given that mild symptoms may not always lead to a clinical consultation [16,29] and given the difficulty of having access to a health professional, which is particularly the case for young adults without important health conditions in Quebec [44]. To counterbalance this possibility, and as done elsewhere [45,46], we used a more sensitive definition for asthma (i.e., asthma-like symptoms) to estimate the prevalence of this disease. The downside of this is the possibility that disease prevalence was overestimated, and that their association with the exposure was underestimated, given some ‘false cases’ were not associated with exposure.

To limit recall bias, but still maintain coherence between studied variables and comparability with previous studies, we used a time interval of 12 months for all our main independent and dependent variables. Nevertheless, one year remains a long time and some respondents may have found it difficult to properly recall the number of events (e.g., number of colds) during this period, thus leading to under-reporting. Finally, a lack of statistical power could hardly explain non-significant associations observed for the entire sample, considering the final sample size and an estimated power of over 0.90 for detecting small effect sizes [47].

A final limit pertains to external validity since our study population was recruited from only one university. Despite the fact that the Université de Sherbrooke’s campuses cover different regions in Quebec, we cannot exclude that geographical (e.g., climate) and social (e.g., housing) contexts are somewhat different from those of other North American universities.

## 5. Conclusions

Our results show that university students, most of whom live as tenants with low income, present a high prevalence of allergic respiratory symptoms. Associations with residential dampness or mold, independent of socio-demographic factors, reinforce its possible role as a causal contributor to various atopic symptoms. This study also adds to the current body of knowledge by suggesting a potential deleterious influence of dampness or mold on daily functioning and academic performance of students living with respiratory diseases. This highlights both health and functional consequences for this serious indoor air hazard. These new findings may provide a window of opportunity to raise awareness on the importance of housing conditions for students among stakeholders in academic and political organizations and to galvanize these parties to act in partnership against insalubrious housing.

## Figures and Tables

**Table 1:** Global prevalence of self-reported atopy and respiratory disease (in the last 12 months) among university students (weighted data, *n* = 1971).

	Prevalence ^1^ (95% CI)
Atopy	
Perceived allergies	33.7% (31.6–35.8)
Atopy diagnosed by a physician	29.0% (27.0–31.0)
Positive skin test for any environmental atopy	19.6% (17.8–21.3)
Positive skin test for mold	5.6% (4.6–6.6)
Atopic respiratory disease	
Allergic rhinitis (SFAR score ≥ 7/16)	32.6% (30.6–34.7)
Asthma-like symptoms (Grassi score ≥ 1/7)	24.0% (22.1–25.8)
Lifetime diagnosed asthma (diagnosed by a physician)	17.2% (15.5–18.9)
Active diagnosed asthma (diagnosed by a physician and Grassi score ≥ 1/7)	10.3% (9.0–11.7)
Uncontrolled diagnosed asthma (≥1 GINA criteria)	5.4% (4.4–6.4)
Burdensome disease among symptomatic subjects	
Burdensome allergic rhinitis (*n* = 628) (VAS ≥ 3/10)	32.4% (28.7–36.0)
Burdensome asthma (*n* = 472) (VAS ≥ 3/10)	11.8% (8.8–14.7)
Respiratory infections	
Upper or lower respiratory bacterial infections	9.6% (8.3–10.9)
Four or more flus or colds	12.0% (10.5–13.4)
Bacterial or viral respiratory infections	19.4% (17.7–21.2)

^1^ Results are weighted for age, sex and campus affiliation.

**Table 2:** Prevalence ^1^ of self-reported respiratory diseases (in the last 12 months) among university students according to student characteristics (weighted data, *n* = 1971).

	Allergic Rhinitis (SFAR Score)		Asthma-Like Symptoms (Grassi Score)		Bacterial or Viral Respiratory Infections	
	% (*n*)	OR (95% CI)	% (*n*)	OR (95% CI)	% (*n*)	OR (95% CI)
Total	32.6% (643)		23.9% (472)		19.4% (383)	
Sex						
Men	27.3% (235)	1	19.7% (170)	1	12.4% (107)	1
Women	36.8% (409)	1.55 (1.28–1.89)	27.2% (302)	1.52 (1.23–1.89)	24.9% (276)	2.34 (1.83–2.98)
Age						
18 to 23 years old	30.6% (242)	1	26.2% (207)	1	19.8% (156)	1
24 to 30 years old	31.8% (182)	1.05 (0.83–1.33)	24.8% (142)	0.93 (0.72–1.19)	20.8% (119)	1.06 (0.81–1.38)
31 years old or more	36.1% (220)	1.28 (1.03–1.61)	20.2% (123)	0.72 (0.56–0.92)	17.6% (107)	0.87 (0.66–1.14)
Parental atopy						
No	23.8% (354)	1	21.0% (312)	1	18.3% (272)	1
Yes	59.8% (290)	4.76 (3.83–5.91)	33.2% (161)	1.87 (1.49–2.35)	22.9% (111)	1.32 (1.03–1.70)
Campus						
Main (Sherbrooke)	28.7% (306)	1	24.8% (265)	1	17.8% (190)	1
Health (Sherbrooke)	31.0% (65)	1.11 (0.81–1.53)	21.9% (46)	0.85 (0.59–1.21)	21.9% (46)	1.29 (0.90–1.85)
Longueuil	34.8% (93)	1.33 (1.00–1.76)	19.5% (52)	0.73 (0.53–1.03)	18.0% (48)	1.02 (0.72–1.45)
Other (Saguenay and off-campus)	41.9% (179)	1.80 (1.42–2.27)	25.5% (109)	1.03 (0.80–1.34)	23.0% (98)	1.37 (1.04–1.81)
Annual family income (CAD)						
Less than $15,000	32.3% (217)	1	25.7% (173)	1	19.3% (130)	1
$15,000 to 24,999	33.9% (107)	1.07 (0.81–1.42)	21.8% (69)	0.81 (0.59–1.11)	13.3% (42)	0.64 (0.44–0.93)
$25,000 to 54,999	36.0% (109)	1.18 (0.89–1.57)	31.9% (97)	1.36 (1.01–1.83)	22.4% (68)	1.19 (0.86–1.66)
$55,000 or more	31.1% (169)	0.94 (0.74–1.20)	18.0% (98)	0.63 (0.48–0.84)	21.9% (119)	1.16 (0.88–1.54)
Refusal or unknown	30.1% (40)	0.91 (0.61–1.36)	26.5% (35)	1.05 (0.69–1.61)	18.0% (24)	0.92 (0.57–1.48)
Smoking (cigarettes)						
Non-smokers	31.8% (500)	1	22.3% (350)	1	19.8% (310)	1
Former smokers	40.1% (89)	1.44 (1.08–1.91)	30.6% (68)	1.46 (1.08–1.99)	14.9% (33)	0.69 (0.47–1.02)
Current smokers	31.2% (54)	0.93 (0.66–1.30)	30.8% (53)	1.46 (1.04–2.06)	23.1% (40)	1.27 (0.87–1.84)
Passive smoking (cigarettes)						
No	33.0% (608)	1	23.7% (437)	1	19.3% (356)	1
Yes	27.9% (29)	0.79 (0.51–1.22)	27.9% (29)	1.24 (0.80–1.94)	20.2% (21)	1.06 (0.65–1.73)
Past experience of insalubrious housing						
No	30.4% (548)	1	21.9% (395)	1	18.5% (334)	1
Yes	56.5% (96)	2.97 (2.16–4.08)	45.3% (77)	2.95 (2.14–4.07)	28.2% (48)	1.73 (1.21–2.46)

^1^ Results are weighted for age, sex and campus affiliation.

**Table 3:** Crude and adjusted association between residential exposure to dampness or mold and self-reported respiratory diseases (in the last 12 months) among university students (unweighted data, *n* = 2097).

	Unexposed	Exposed	Crude OR	Adjusted OR ^1^	Adjusted OR ^2^
	% (*n*)	% (*n*)	(95% CI)	(95% CI)	(95% CI)
Respiratory disease					
Allergic rhinitis (*n* = 2023)	28.9% (375)	35.5% (258)	1.35 (1.11–1.64) **	1.25 (1.01–1.55) *	1.30 (1.05–1.60) *
Asthma-like symptoms (*n* = 2071)	21.8% (288)	33.1% (248)	1.78 (1.45–2.17) ***	1.70 (1.37–2.11) ***	1.75 (1.42–2.16) ***
Respiratory infections (*n* = 1999)	20.2% (229)	21.4% (153)	1.08 (0.86–1.35)	1.07 (0.85–1.36)	1.07 (0.85–1.35)
Impact of respiratory disease (among symptomatic students only)					
Burdensome allergic rhinitis (*n* = 616)	27.5% (100)	40.3% (102)	1.78 (1.26–2.50) ***	1.75 (1.22–2.50) **	1.77 (1.25–2.50) **
Burdensome asthma (*n* = 520)	7.6% (21)	16.1% (39)	2.35 (1.34–4.12) **	^3^	2.34 (1.31–4.16) **
Uncontrolled asthma (*n* = 221)	44.7% (55)	64.3% (63)	2.23 (1.29–3.84) ***	^3^	2.17 (1.21–3.88) **
Decreased academic performance (*n* = 1026)	6.5% (40)	10.6% (43)	1.70 (1.09–2.67) *	^3^	1.76 (1.24–2.50) **

* *p* value < 0.05; ** *p* value < 0.01; *** *p* value < 0.001. ^1^ Adjusted for: sex, age (three categories), campus (four categories), smoking status (three categories), parental atopy, past experience of insalubrious housing, annual family income (five categories); ^2^ Adjusted for: sex, age (three categories), smoking status (three categories), parental atopy, past experience of insalubrious housing, annual family income (two categories); ^3^ Group sample too small.
